# Modeling and Experimental Investigation of the Impact of the Hemispherical Tool on Heat Generation and Tensile Properties of Dissimilar Friction Stir Welded AA5083 and AA7075 Al Alloys

**DOI:** 10.3390/ma17020433

**Published:** 2024-01-16

**Authors:** Ahmed R. S. Essa, Ramy I. A. Eldersy, Mohamed M. Z. Ahmed, Ali Abd El-Aty, Ali Alamry, Bandar Alzahrani, Ahmed E. El-Nikhaily, Mohamed I. A. Habba

**Affiliations:** 1 Mechanical Department, Faculty of Technology and Education, Suez University, Suez 43211, Egypt; ahmed.eessa@suezuniv.edu.eg (A.R.S.E.); iramy3791@gmail.com (R.I.A.E.); ahmed.eassa@ind.suezuni.edu.eg (A.E.E.-N.); mohamed.atia@suezuniv.edu.eg (M.I.A.H.); 2 Faculty of Engineering, King Salman International University, El Tor 45615, Egypt; 3 Department of Mechanical Engineering, College of Engineering at Al Kharj, Prince Sattam Bin Abdulaziz University, Riyadh 11942, Saudi Arabia; moh.ahmed@psau.edu.sa (M.M.Z.A.); a.alamry@psau.edu.sa (A.A.); ba.alzahrani@psau.edu.sa (B.A.); 4 Department of Metallurgical and Materials Engineering, Faculty of Petroleum and Mining Engineering, Suez University, Suez 43211, Egypt; 5 Mechanical Engineering Department, Faculty of Engineering, Helwan University, Cairo 11795, Egypt

**Keywords:** friction stir welding, hemispherical tool, peak temperature, dissimilar aluminum alloys, mechanical properties

## Abstract

This study investigated the effect of a hemispherical friction stir welding (FSW) tool on the heat generation and mechanical properties of dissimilar butt welded AA5083 and AA7075 alloys. FSW was performed on the dissimilar aluminum alloys AA5083-H111 and AA7075-T6 using welding speeds of 25, 50, and 75 mm/min. The tool rotation rate was kept constant at 500 rpm. An analytical model was developed to calculate heat generation and temperature distribution during the FSW process utilizing a hemispherical tool. The experimental results were compared to the calculated data. The latter confirms the accuracy of the analytical model, demonstrating a high degree of agreement. Sound FSW dissimilar joints were achieved at welding speeds of 50 and 25 mm/min. Meanwhile, joints created at a welding speed of 75 mm/min exhibited a tunnel-like defect, which can be attributed to the minimal heat generated at this particular welding speed. At a lower welding speed of 25 mm/min, a higher tensile strength of the dissimilar FSWed joints AA5083 and AA7075 was achieved with a joint efficiency of over 97%.

## 1. Introduction

Since its invention in 1991, friction stir welding (FSW) has gained popularity for joining different aluminum alloys (AA), particularly difficult-to-weld alloys such as the 2xxx and 7xxx series [1]. FSW parameters, tool design, and tool materials are continuously updated. Process operation, including the development of the bobbin tool [2,3,4,5,6], stationary shoulder [7,8,9], reverse dual rotation [10], and others, has also progressed. Recent advancements in solid-state welding have made FSW one of the best methods for joining various alloys, particularly dissimilar alloys. Several process parameters control the FSW of dissimilar alloys. The impact of various factors on the characteristics of friction stir-welded joints between dissimilar aluminum alloys has been the subject of numerous studies [8,9,10,11,12]. Tool geometry and design have attracted the attention of many researchers because of the vital role that the tool plays in the quality of friction stir welded joints [13,14,15,16,17,18,19]. According to Raj Kumar et al. [20], FSW of dissimilar aluminum alloys AA5052 and AA6061 is affected by welding settings and tool design. They reported that the alloys may be joined effectively and with good bonding using cylindrical threaded pins. Compared with other tools, the stepped pin tool provided the maximum tensile strength for the welded joints, and generated a sound joint at welding speeds of 1000 rpm and 40 mm/min. A study [1] on the effect of pin eccentricity on the characteristics of friction stir welded AA7075 found that a clear impact on the tensile characteristics of the welded joints; a pin eccentricity of 0.2 mm is the ideal value for maximizing strength and elongation. 

In the FSW process, the heat generated is assumed to be a combination of two sources: (i) the friction between the tool and the workpiece and (ii) the plastic shear deformation of the weld nugget in the vicinity of the pin [21,22,23,24,25]. The tool contacts the materials and generates the needed heat through three regions: the shoulder, the pin side, and the pin tip, so the friction between the pin side and the joint material is an important source of heat. Moreover, increasing the pin side area will greatly affect the share of every part of the FSW tool in generating heat by friction [26,27,28,29,30]. Sadoun et al. [31] studied the effect of pin side area on heat generation and macrostructure developed during friction stir welding 5 mm thick AA7075 joints. They reported that the temperature distribution is influenced by the ratio of the pin side area, as it directly impacts the quantity of heat created by the pin side. Therefore, a tool with a higher ratio of pin side area is favored from a microstructure perspective. Employing a tool equipped with a semi-spherical pin and a high pin side area ratio (29.83%) results in a joint characterized by a smaller grain size and greater tensile strength. The joint soundness and quality of dissimilar welding of AA7075-T6 and AA5083-H111 depend mainly on the placement of AA7075 (higher flow stress alloy), which needs a higher heat input than AA5083 [32]. The joint efficiency is also higher at higher heat inputs (low travel speeds). The present work aims to increase the contact area of the pin side by using a modified tool design (hemispherical tool) and to investigate its effect on the properties of dissimilar AA7075/AA5083 friction stir welded joints. An analytical model for heat generation will also be modified according to the current tool design.

## 2. Materials and Methods

### 2.1. Material Description

A friction stir welding process was used to create dissimilar joints between the aluminum alloys AA5083-H111 and AA7075-T6, utilizing FSW equipment for testing and development. Table 1 and Table 2 list the chemical composition and mechanical characteristics of each alloy. The composition of the AA5083-H111 and AA7075-T6 plates was determined using Foundry-Master Pro (Oxford Instruments, Abingdon, UK). The weld samples were created using two 5 mm thick, 100 mm wide, and 150 mm long plates.

### 2.2. FSW Procedures

**Figure 1:**
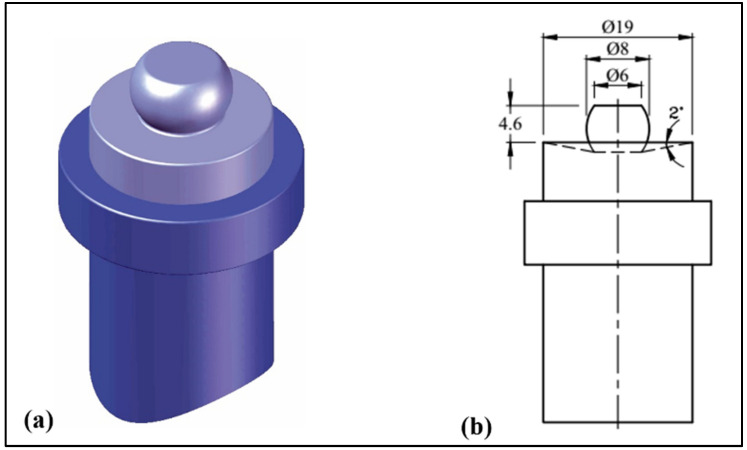
FSW tool (**a**) shape and (**b**) dimensions.

The welding tools were made from a 30 mm diameter W302 cold-worked tool steel rod (Bühler AG, Branch office Cairo, Cairo, Egypt), that had been heat-treated to HRC62, with the following chemical composition: 0.39% C, 0.1% Si, 0.40% Mn, 5.2% Cr, 0.95% V, 1.4% Mo, and 90.6 wt% Fe. As illustrated in Figure 1, a hemispherical tool pin was made with an 8 mm diameter, 4.6 mm long, and a smooth 19 mm diameter shoulder. The pin also has a 2° concavity.

A constant tool plunge depth (shoulder penetration) of 0.2 mm for all experiments was used, and a tilt angle of 3° applied. The peak temperatures were measured on the top surface of the advancing side of the weld joints using a laser thermometer (Quicktemp 8630-T3 device, Testo Company, Berlin, Germany) that has a measurement error range from 1° to 12° based on the object emissivity. The plunge forces were recorded by the FSW machine for research and development. Table 3 lists the applied FSW to produce AA5083-H111 and AA7075-T6 dissimilar joints.

The metallographic samples were cut perpendicular to the welding direction. The samples were ground, polished, and etched by Keller’s chemical agent (2 mL HF, 3 mL HCL, 5 mL nitric acid, and 190 mL water) for 25 s to reveal the microstructure of the weld zones. The grain size developed microstructures were measured using ImageJ software (Version 1.54d, Wayne Rasband and contributors, National Institutes of Health, Bethesda, MD, USA). The Vickers hardness measurements were done using a Vickers Hardness Tester type HWDV-75 (TTS Unlimited, Osaka, Japan). The measurements were made at the center of the joint on the cross-sections perpendicular to the direction of the FSW path. Tensile test specimens were obtained in a direction perpendicular to the welding direction and were prepared in accordance with the ASTM E8/E8M-16a standard. At room temperature and 0.02 mm/s crosshead speed, a universal tensile testing machine (Instron 4208, 30-ton capacity, Norwood, MA, USA) performed tensile tests.

## 3. Heat Generation Estimation Model

**Figure 2:**
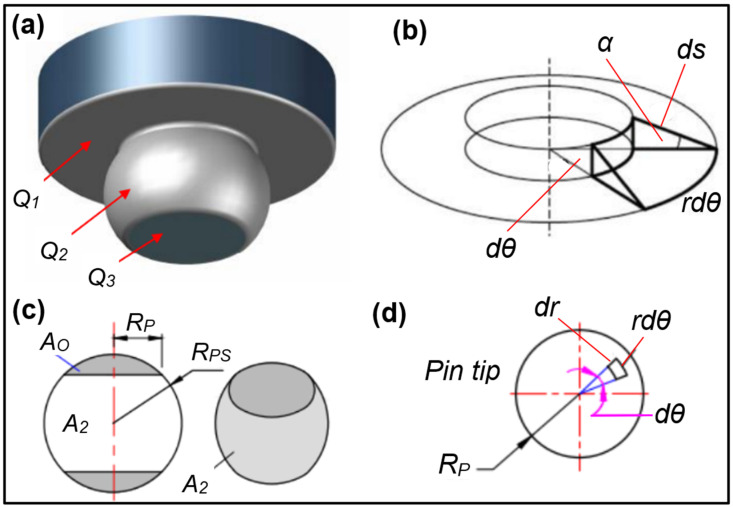
Schematic drawing of surface orientations and infinitesimal segment areas: (**a**) the different heat generation regions, (**b**) concave shoulder, (**c**) pin side, and (**d**) pin tip.

An analytical model for heat generation during friction stir welding was created using a hemispherical pin profile. The suggested model is a revision of earlier analytic models [33,34]. Figure 2a shows the tool design with a hemispherical pin profile and illustrates the three areas where heat is anticipated to be produced by friction. *Q*_1_ is the heat produced by the concave shoulder, *Q*_2_ by the hemispherical side of the pin, and *Q*_3_ by the pin tip, resulting in the overall heat generation, which is given by Equation (1) as:

*Q_total_* = *Q*_1_ + *Q*_2_ + *Q*_3_ (1)

For the current analytical modeling, some assumptions were taken into account. First, the analytical estimation based on the supposition that the contact shear stress is uniform was considered. Second, the sliding state of the shearing occurs at the contact interface. Third, other heat-generating mechanisms, such as deformation, were not considered. Figure 2 depicts the hemispherical tool and workpiece contact surface determined by position and orientation in relation to the rotational axis.

The current analytical modeling, a modified version of the analytical model provided by Essa et al. [35] and Schmidt et al. [36], utilized a straightforward tool design with a concave shoulder surface, a hemispherical pin, and a flat pin surface. The radius of the sphere shape *Rps* and the radius of the pin tip *Rp* define the hemispherical pin surface and the concave shoulder surface, respectively. Despite being different, the formulas for each surface area orientation are based on the fundamental equation for heat generation [37]:

(2) $$dQ=\omega dM=\omega \cdot r\cdot dF=\omega \cdot r\cdot \tau_{contact}dA$$

### 3.1. Heat Generated from the Shoulder Surface

To calculate the heat produced by the concave shoulder surface spinning around the tool axis, an infinitesimal segment on that surface was taken into account. This infinitesimal segment region is subject to uniform contact shear stress, as seen in Figure 2b. This section only makes a tiny force and torque contribution. The heat produced by this section is:

(3) $$dQ_{1}=\omega \cdot r\cdot \tau_{contact}rd\theta ds$$

where *r* is the distance between the considered area and the center of rotation, *ω* is the angular velocity, and “dθ, ds” are the segment dimensions. Integration of Equation (3) over the concave shoulder area from *Rp* to *Rs* gives the shoulder heat generation, *Q*_1_, as follows:

(4) $$dQ_{1}=\omega \cdot r^{2}\cdot \tau_{contact}\cdot d\theta \cdot \frac{dr}{\cos\alpha }$$

(5) $$Q_{1}=\int_{0}^{2\pi }\int_{R_{p}}^{R_{s}}\omega \cdot r^{2}\cdot \tau_{contact}\cdot d\theta \cdot \frac{dr}{\cos\alpha }$$

(6) $$Q_{1}=2\pi \cdot \omega \cdot \tau_{contact}\cdot \frac{\left({R_{s}}^{3}-{R_{p}}^{3}\right)}{3\cos\alpha }$$

### 3.2. Heat Generated from the Hemispherical Surface

The pin consists of a hemispherical surface with a radius of sphere *Rps* and radius of pin tip *Rp* and pin height *Hp*. The heat generated from the pin side is given by the following equations over the pin side area.

(7) $$dQ_{2}=\omega \cdot r\cdot \tau_{contact}\cdot dA_{2}$$

(8) $$Q_{2}=\int \omega \cdot r\cdot \tau_{contact}\cdot dA_{2}=\omega \cdot R_{PS}\cdot \tau_{contact}\cdot A_{2}$$

(9) $$A_{2}=A_{spher}-{2A}_{O}$$

(10) $$A_{2}=4\pi R_{PS}^{2}-\left[2\int_{0}^{2\pi }\int_{0}^{r_{p}}\sqrt{1+\frac{r^{2}{cos}^{2}\theta +r^{2}{sin}^{2}\theta }{{r_{PS}^{2}-r}^{2}{cos}^{2}\theta -r^{2}{sin}^{2}\theta }}rdrd\theta \right]$$

(11) $$A_{2}=4\pi R_{PS}^{2}-\left[4\pi R_{PS}^{2}-4\pi R_{PS}\sqrt{R_{PS}^{2}+R_{P}^{2}}\right]$$

(12) $$A_{2}=4\pi R_{PS}\sqrt{R_{PS}^{2}+R_{P}^{2}}$$

(13) $$Q_{2}=\omega \cdot R_{PS}\cdot \tau_{contact}\cdot 4\pi R_{PS}\sqrt{R_{PS}^{2}+R_{P}^{2}}$$

(14) $$Q_{2}=4\omega \cdot \pi R_{PS}^{2}\cdot \tau_{contact}\cdot \sqrt{R_{PS}^{2}+R_{P}^{2}}$$

### 3.3. Heat Generation from the Pin Tip Surface

The following equations give the heat generated from the pin tip. Assuming that the flat pin tip gives the heat generated from the pin tip, *Q*_3_, thus:

(15) $$dQ_{3}=\omega \cdot r\cdot \tau_{contact}\cdot rd\theta dr$$

(16) $$Q_{3}=\int_{0}^{2\pi }\int_{0}^{R_{p}}\omega \cdot r\cdot \tau_{contact}\cdot rd\theta dr=\frac{2}{3}\pi \omega \cdot \tau_{contact}\cdot R_{p}^{3}$$

From Equations (6), (14) and (16), *Q_Total_* can be calculated as written in Equation (17),

(17) $$Q_{Total}=Q_{1}+Q_{2}+Q_{3}$$

(18) $$Q_{Total}=\left(2\pi \omega \cdot \tau_{contact}\frac{\left(R_{s}^{3}-R_{p}^{3}\right)}{3\cos\alpha }\right)+\left(4\omega \cdot \pi R_{PS}^{2}\cdot \tau_{contact}\cdot \sqrt{R_{PS}^{2}+R_{P}^{2}}\right)\;+\left(\frac{2}{3}\pi \omega \cdot \tau_{contact}\cdot R_{p}^{3}\right)$$

(19) $$Q_{Total}=\frac{2}{3}\pi \omega \cdot \tau_{contact}\cdot \left[\left(\frac{\left(R_{s}^{3}-R_{p}^{3}\right)}{\cos\alpha }\right)+\left(6R_{PS}^{2}\sqrt{R_{PS}^{2}+R_{P}^{2}}\right)+\left(R_{p}^{3}\right)\right]$$

The estimated shear stress for the sliding condition is given by:

(20) $$\tau_{contact}=p\cdot \mu$$

and pressure “*p*” is given by:

(21) $$p=F/\pi \cdot {R_{s}}^{2}$$

The energy per unit length can be calculated by dividing Equation (19) by the welding speed. Thus:

(22) $$Q_{Energy/Length}=\frac{2\pi \omega F\mu }{3vR_{S}^{2}}\left[\left(\frac{\left(R_{s}^{3}-R_{p}^{3}\right)}{\cos\alpha }\right)+\left(6R_{PS}^{2}\sqrt{R_{PS}^{2}+R_{P}^{2}}\right)+\left(R_{p}^{3}\right)\right]$$

The coefficient of friction (*µ*) varies with temperature [38,39]. However, in the present model, for demonstration purposes, it was considered to be 0.5. The effective energy per weld length (*Q_Eff_*) [40,41,42] is defined as the energy per weld length multiplied by the transfer efficiency (“*β*” ratio of the pin length “*Hp*” to the workpiece thickness “*t*”) and given by

(23) $$Q_{Eff}=\beta \cdot Q_{Energy/Length}=\left(H_{p}/t\right)\times Q_{Energy/Length}$$

The analytical relationship developed by Hamilton et al. [40] between the temperature ratio and the effective energy level *Q_Eff_* was considered. The analytical equation is given by Equation (24) as:

(24) $$\frac{T_{max}}{T_{S}}=1.56\times 10^{-4}Q_{Eff}+0.54$$

The current model should provide an upper bound for the thermal profiles. The finite element (FE) heat flux can be related to the radial position r by using an equation developed by Khandkar et al. [41] to give

(25) $$\dot{Q}\left(r\right)=Q_{Eff}\cdot \;r/\left(\frac{2}{3}\pi R_{S}^{3}+2\pi R_{PS}^{2}H\right)$$

where $\dot{Q}\left(r\right)$ is the local heat flux and is linearly related to r. This FE equation has been used to calculate the moving heat input at the three different interfaces between the tool and the workpiece. Alternatively, the FE heat flux can also be related to r through tool rotation and the average shear stress to yield the same effect. The energy balance equation of the FSW process can be described by:

(26) $$\rho c\dot{T}=k\left(\frac{\partial^{2}T}{\partial x^{2}}+\frac{\partial^{2}T}{\partial y^{2}}+\frac{\partial^{2}T}{\partial z^{2}}\right)+\dot{Q}$$

where *T* is the temperature, $\dot{T}$ is the rate of change in temperature, *c* is the specific heat, ρ is the density, *k* is the thermal conductivity, and $\dot{Q}$ is the rate of moving heat generation per unit volume.

(27) $$-k\left(\frac{\partial T}{\partial x}\right)=\alpha_{sim}\left(T_{x}-T_{0}\right)$$

where $\alpha_{sim}$ is the convection heat transfer coefficient, and its value in this paper is 15 W m^−2^ K^−1^ with an ambient temperature $T_{0}$ of 24 °C for the top and side surfaces of the workpiece. 

**Figure 3:**
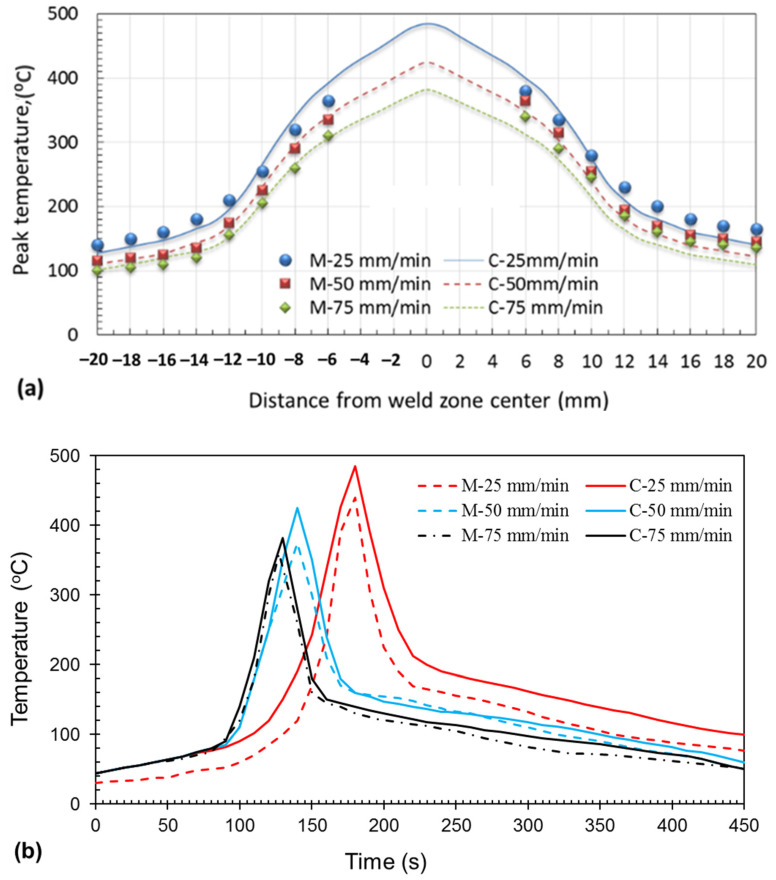
(**a**) Variation of calculated and experimental temperature for FSWed joints at 25, 50, and 75 mm/min welding speeds. M: refers to “measured” and C: refers to “calculated”, (**a**) temperature distribution away from the weld joint center line, (**b**) The experimental and calculated temperature history.

To validate the proposed model, experimental and simulated thermal profiles are presented in this section. Figure 3a shows the experimentally measured spatial temperature distribution for the three welding speeds of 25, 50, and 75 mm/min, whereas Figure 3b shows the experimental temperature history. Good agreement is observed between the experimental and calculated values.

The peak temperature at any distance from the weld zone center increased with decreasing traveling speed because of increasing heat input with decreasing traveling speed. Additionally, the maximum peak temperature was obtained at the center of the nugget zone at the three welded joints, which may be caused by the severe plastic deformation that results in a high quantity of heat input. Another dramatic observation is that a higher value of the peak temperatures was observed in the advancing side where the alloy AA5083 was placed compared with the retreating side where the alloy AA7075 was placed, and this may be attributed to the increasing rotation speed in the advancing side compared with the retreating side, causing the advancing side to be hotter [42]. The results of the proposed model indicate an increase in the heat generated at a 25 mm/min travel speed.

## 4. Evaluation of FSWed AA7075/AA5083 Dissimilar Joints

The results of the mechanical properties of the welded joints will be discussed with macrostructure and microstructure support. The heat generation during FSW and the temperature ratio will also be analyzed based on the proposed model and experimental measurements.

### 4.1. Microstructure Investigations

**Figure 4:**
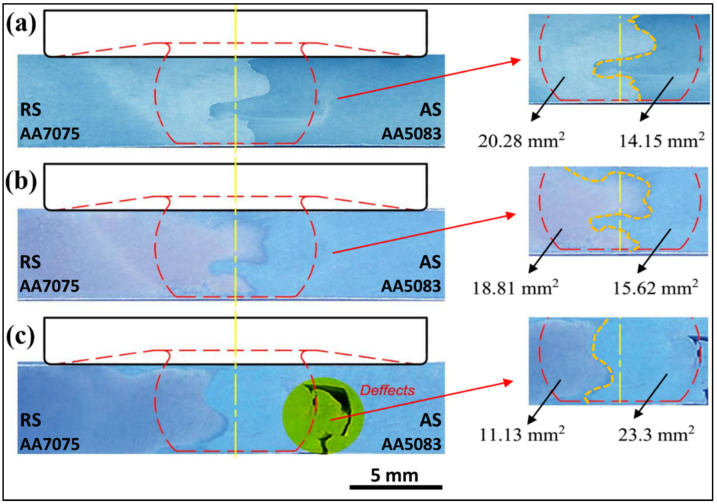
Macrostructure of dissimilar FSWed joints AA7075 and AA5083 at different welding speeds: (**a**) 25 mm/min, (**b**) 50 mm/min, and (**c**) 75 mm/min.

The macrostructure for the cross-section of the FSW 5083/7075 dissimilar joints is shown in Figure 4. A clear interface is observed in the nugget zone for all three joints. Another important observation is the superior adhesion and soundness obtained in the joints welded at travel speeds of 25 and 50 mm/min, but a tunnel defect is formed in the joint welded at a travel speed of 75 mm/min. The formation of tunnel defects has been researched by several authors [34,35,36,37,38,39,40,41,42,43,44,45], especially for friction stir welding of dissimilar aluminum alloys. The configuration of the welded joints plays an important role in the formation of defects [46] and depends mainly on the selection of the alloy to be placed on the advancing side and on the retreating side. In the present work, the weaker-strength AA5083 alloy was placed on the advancing side, and the higher-strength AA7075 alloy was placed on the retreating side. The defect was formed on the advancing side where the 5083 alloy was placed. In [47], it was concluded that defects will exist in welded joints if a higher-strength alloy is placed on the advancing side, which disagrees with the results obtained in the present work. On the other hand, since the material on the advancing side of the weld is flowing in the same direction as the tool, this can cause the material to become more fragmented and less cohesive, making it more susceptible to defect formation. Also, the material on the advancing side of the weld is subjected to more intense frictional heating than the material on the retreating side. This can cause the material to overheat and melt, making it more susceptible to defect formation regardless of the alloy type, which agrees with the results of the present work, where the defect was formed on the advancing side [48,49]. Moreover, AA5083 is a highly deformation-resistant alloy and has a high flow strength, so increasing the travel speed results in low heat input, which may cause poor intermixing between the two alloys, increasing the likelihood of defects [50]. The contribution of each alloy band was calculated approximately, as shown in Figure 4. At a travel speed of 25 mm/min, the band area of AA7075 was approximately 20.28 mm^2^, higher than that of the band area of AA5083 (14.15 mm^2^), whereas at a travel speed of 75 mm/min, the contribution of AA 5083 to the formation of the nugget area was higher (for AA 5083, the band area was 23.mm^2^ and 11.13 mm^2^ for AA 7075).

**Figure 5:**
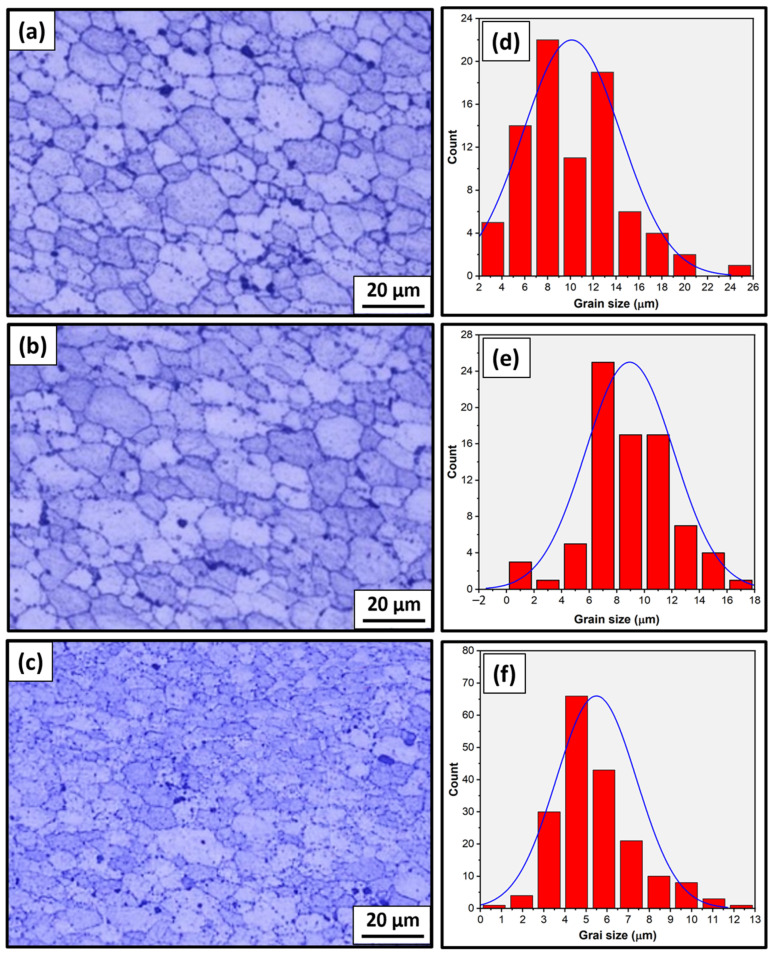
Microstructure and grain size analysis of dissimilar FSWed joints AA7075 and AA5083 at different welding speeds of (**a**,**d**) 25, (**b**,**e**) 50, and (**c**,**f**) 75 mm/min.

Optical microstructure investigations are paramount for FSW joints; examination of the microstructure features with grain size indicates the expected mechanical properties of welded joints. Figure 5 shows the optical microstructures and their grain size analysis of the weld zone for the dissimilar AA7075/AA5083 welded joints produced using FSW travel speeds of 25 (Figure 5a,d), 50 (Figure 5b,e), and 75 mm/min (Figure 5c,f). The microstructures of the dissimilar FSWed joints obtained fine equiaxed grains in the weld zone, as depicted in Figure 5a–c. During the FSW process, the intense localized heat generated by the rotating tool (stirring action) induces severe plastic deformation and intense stirring action in the stir zone. This action (stirring process) breaks down the coarse grains of the initial plates and promotes the formation of fine, equiaxed grains. The grain size analysis of the developed microstructures of the dissimilar welded joints revealed the significant effects of travel speeds on the grain size of the developed microstructures in the weld zone. Decreasing the travel speed from 75 to 25 mm/min tended to result in a finer grain structure within the weld zone (Figure 5d–f). The higher travel speed of 75 mm/min corresponds to a shorter exposure time to the elevated temperatures generated during the FSW process. The smallest grain size of 4.48 µm (average grain size) in the weld zone was developed in the dissimilar AA75075/AA5083 joint welded at a travel speed of 25 mm/min, while the travel speed of 75 mm/min resulted in an average grain size of 10.12 µm, as shown in Figure 5d–f. This difference in the sizes of grain structures in the weld zone is expected to affect the mechanical properties of the welded joints, such as the tensile and hardness properties.

### 4.2. Mechanical Properties

**Figure 6:**
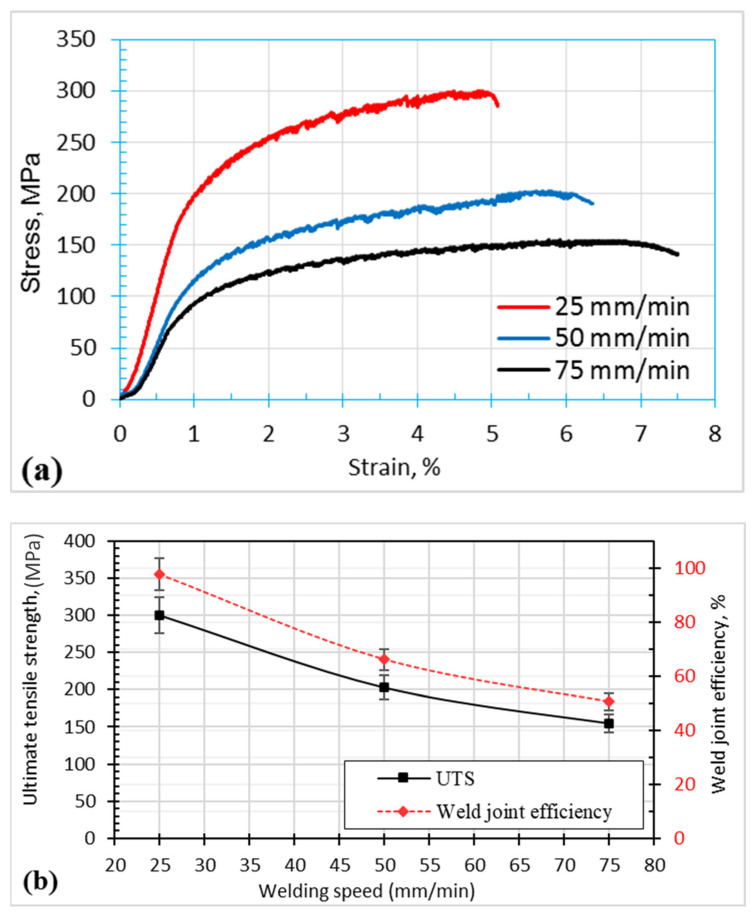
Tensile properties of dissimilar FSWed joints AA7075–AA5083 at different welding speeds of 25, 50, and 75 mm/min. (**a**) Stress-Strain curves; (**b**) ultimate tensile strength and welding joint efficiency.

**Figure 7:**
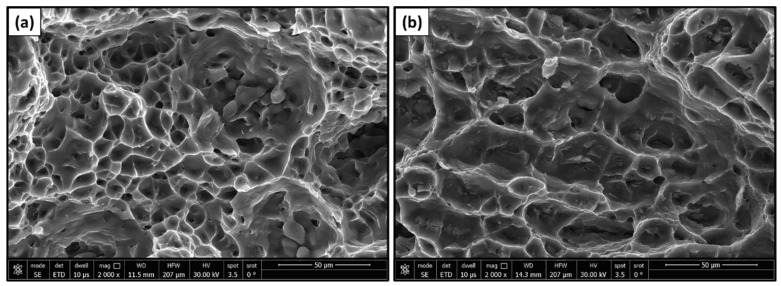
Fracture surface morphology of the dissimilar AA7075/AA5083 FSWed joints welded at travel speeds of (**a**) 25 mm/min and (**b**) 75 mm/min.

Figure 6a illustrates stress-strain curves for joints created at welding speeds of 25, 50, and 75 mm/min, while Figure 6b illustrates the impact of travel speed on the ultimate tensile strength and joint efficiency of dissimilar FSWed butt joints welded at various travel speeds of 25, 50, and 75 mm/min at a constant rotational speed of 500 rpm (b). When the travel speed decreases, the tensile strength of the welded connection increases, since a higher heat input is possible at slower travel rates. When using a travel speed of 25 mm/min, a maximum joint efficiency of 97.7% was attained. This may be attributed to the enhancement of the bond strength [51,52], and the increase in the band area of 7075 (high strength) in the nugget zone compared with that of 5983, as shown in Figure 4. A similar work, using a different tool with an 18 mm diameter concave shoulder and a 4.8 mm long unthreaded tapered cylindrical pin, reported similar results with a maximum joint efficiency of approximately 90% at the lowest travel speed (50 mm/min) [53]. The higher weld joint efficiency in the present work compared with other similar works may be attributed to the effect of the novel tool design (hemispherical pin tool), which may result in an increase in the contact area between the tool and the joint that causes severe deformation of the stir zone, resulting in a finer structure. Additionally, some scholars used different tool designs (threaded, squared, stepped, cylindrical, and tapered) to friction stir weld dissimilar AA 5052 and AA 2024 [49]. They concluded that the maximum tensile percentage or joint efficiency of 90% is obtained using the stepped tool. More recent work [53] used a triangular threaded pin profile for FSW of dissimilar 1050-H14 and 5083-H111 aluminum alloys, and the maximum tensile percentage was 63.4%. Figure 7 shows the fracture surface of the tensile tested specimens for the dissimilar FSWed joints produced at travel speeds of (a) 25 mm/min and (b) 75 mm/min. The FSW joint produced at a 25 mm/min travel speed revealed an equiaxed deep and shallow dimple smaller than those detected for the joint welded at a 75 mm/min travel speed (Figure 7a,b). This observation confirms the higher tensile strength of the FSWed joints produced at a low travel speed of 25 mm/min than those welded at a travel speed of 75 mm/min (Figure 6).

**Figure 8:**
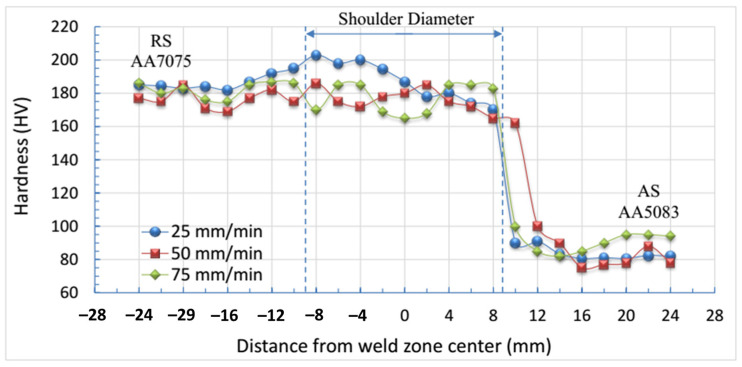
Hardness profiles of dissimilar FSWed joints AA7075 and AA5083.

Figure 8 illustrates the hardness profile for the welded joints produced at different traveling speeds of 25, 50, and 75 mm/min and a constant rotation speed of 500 rpm. It can be observed that the weld zone of the dissimilar joints revealed higher hardness than the AA5083 initial materials, where the highest hardness value of 200 HV was discovered at the lowest travel speed of 25 mm/min. Furthermore, based on the hardness profile in Figure 8, it is evident that the hardness profile exhibits three distinct zones. The zone with the lowest hardness corresponds to the AA5083 side (advancing side), while the zone with the highest hardness corresponds to the AA7075 side (retreating side). The dissimilar weld zone (AA7075/AA5083) displayed hardness values that fell within the range of the other two zones, according to the experimental temperature of the dissimilar welded joints ranging from 385 to 487 °C. It has been established that the amount of heat generated depends on various factors, including the rotating speed, applied pressure, frictional conditions, and tool design. Conversely, heat dissipation is contingent upon various factors, such as the pace at which welding is conducted, the thickness of the material being welded, and the prevailing atmospheric conditions. The pace at which heat is generated and dissipated plays a crucial role in determining the extent of the temperature increase and subsequent structural modifications in the weld zone and its surrounding materials. This leads to the creation of a supersaturated solid solution within the heat-affected zones and an averaging condition in the surrounding material [47,48]. The observed changes in hardness values along the cross-section of the dissimilar joints can be attributed mostly to the structural modifications that occur.

### 4.3. Heat Generation and Temperature Profiles

**Figure 9:**
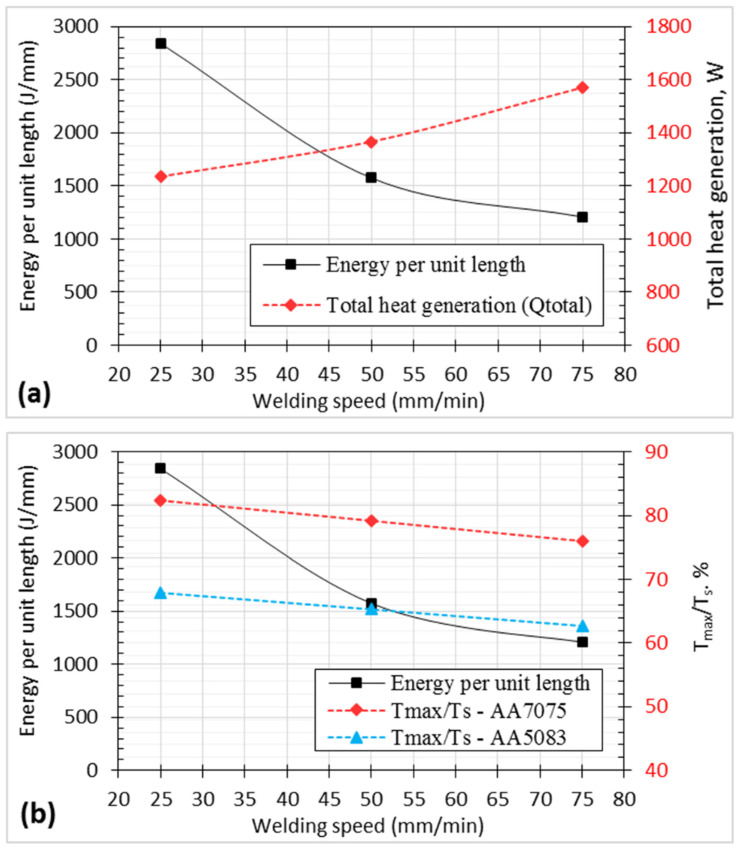
Effect of welding speed on the heat generation and temperature ratio for dissimilar weld joints AA 7075 and AA 5083: (**a**) effective energy per unit length and total heat generation, (**b**) effective energy per unit length and temperature ratio.

The above-described analytical model to calculate the heat generated was utilized to better understand the impact of a hemispherical tool on the characteristics of friction stir welded connections. Figure 9a illustrates a significant link between the welding speed and heat output based on the proposed and validated models above. The relationship between the total heat produced and the energy per unit length (calculated by dividing the total heat produced by the welding speed) is very strong. It is assumed that the energy per unit length decreases when the welding speed increases. On the other hand, the total heat generated increases with increasing welding speed, which may be attributed to the increase in plunge force and torque that cause the increases in friction dissipation energy generated [50,52]. Higher tensile strength and joint efficiency were obtained at a lower welding speed (Figure 5b), which means that the higher the energy per unit length is, the stronger the weld joint becomes.

The effect of the welding speed on the temperature ratio (*T_max_*/*Ts*), where *T_max_* is the maximum temperature at the nugget zone, and *Ts* is the melting point for the alloy, is plotted in Figure 9b. It is clear that both energy per unit length and *T_max_*/*Ts* ratio decrease with increasing travel speed. It is important to note that the temperature ratio for AA 7075 exceeds 75% at all traveling speeds, whereas the temperature ratio for AA5083 reaches a maximum value of 68% at a 25 mm/min travel speed and 62% at a 75 mm/min travel speed. This means that the heat input available for sound joint formation is slightly lower for the 5083 alloy than for the 7075 alloy. This may be an important concept for tunnel defect formation when the temperature ratio decreases [44,45].

## 5. Conclusions

A hemispherical pin tool was used for friction stir welding of dissimilar 5083 and 7075 aluminum alloys using a constant rotation speed of 500 rpm and different travel speeds of 25, 50, and 75 mm/min. An analytical model was proposed and validated experimentally. Good agreement was observed between the proposed model of heat energy generated with the associated peak temperatures and the experimentally obtained results. The energy per unit length is a more important index than the total generated heat since tensile strength and joint efficiency become optimized as energy per unit length increases. Defect-free joints were produced with travel speeds of 25 and 50 mm/min. A tunnel defect was formed on the advancing side where the 5083 alloy was placed, using a travel speed of 75 mm/min. A maximum tensile percentage of 97.7% was obtained at a travel speed of 25 mm/min. The hemispherical pin tool is suitable for friction stir welding processes and needs more attention in future work.

## Figures and Tables

**Table 1:** Chemical composition of the present aluminum alloys.

Type	Element (wt.%)								
	Si	Fe	Cu	Mn	Mg	Cr	Zn	Ti	Al
5083	0.04	0.15	0.02	0.56	4.75	0.05	0.04	0.05	rest
7075	0.07	0.21	1.94	0.05	2.66	0.21	5.94	0.01	rest

**Table 2:** Mechanical properties of the base aluminum alloys.

Alloy	Tensile Strength (MPa)	Proof Stress 0.2% (MPa)	Elongation (%)
AA5083-H111	307	156	19
AA7075-T6	571	495	11.5

**Table 3:** The FSW process parameters.

Welding Parameters	Welding Speed (mm/min)	Rotation Speed (rpm)
1	25	500
2	50	500
3	75	500

## Data Availability

Data will be made available upon request through the corresponding author.
